# Antigen-Presenting Cell Isolevuglandins Link Salt Sensitivity of Blood Pressure to Insulin Resistance

**DOI:** 10.1210/clinem/dgaf556

**Published:** 2025-10-07

**Authors:** Lale A Ertuglu, Mert Demirci, Ashley L Mutchler, Cheryl L Laffer, Mohammad Saleem, T Alp Ikizler, Annet Kirabo

**Affiliations:** Department of Medicine, Vanderbilt University Medical Center, Nashville, TN 37232, USA; Department of Medicine, Vanderbilt University Medical Center, Nashville, TN 37232, USA; Department of Biomedical Sciences, School of Graduate Studies, Meharry Medical College Nashville, Nashville, TN 37208, USA; Department of Medicine, Division of Clinical Pharmacology and Genetic Medicine, Vanderbilt University Medical Center Nashville, Nashville, TN 37232, USA; Department of Medicine, Division of Clinical Pharmacology and Genetic Medicine, Vanderbilt University Medical Center Nashville, Nashville, TN 37232, USA; Department of Medicine, Division of Nephrology, Vanderbilt University Medical Center, Nashville, TN 37232, USA; Department of Medicine, Division of Clinical Pharmacology and Genetic Medicine, Vanderbilt University Medical Center Nashville, Nashville, TN 37232, USA

**Keywords:** salt sensitivity, hypertension, insulin resistance, oxidative stress

## Abstract

**Context:**

Insulin resistance has been associated with salt sensitivity and low sodium intake; however, the mechanisms remain elusive. Our previous studies showed that sodium-induced isolevuglandin (IsoLG) formation in antigen-presenting cells (APCs) leads to systemic inflammation and that salt-sensitive hypertension and IsoLG formation in APCs are affected by acute alterations in salt intake in salt-sensitive but not salt-resistant people.

**Objective:**

In this clinical study, we investigated how acute salt loading and depletion change insulin resistance markers and whether these changes are linked with changes in IsoLGs in APCs.

**Methods:**

A total of 20 participants with hypertension underwent an inpatient protocol of salt loading and depletion for assessment of salt sensitivity. Plasma glucose and insulin levels were measured after 24 hours of salt loading and depletion and insulin resistance was measured by the homeostasis model assessment index (HOMA-IR). IsoLG-adduct accumulation in APCs (dendritic cells, classical, intermediate and nonclassical monocytes) was assessed by flow cytometry.

**Results:**

Baseline insulin resistance correlated with higher salt sensitivity. Insulin resistance significantly increased from salt loading to salt depletion. Salt depletion induced changes in IsoLG + APCs significantly correlated with changes in HOMA-IR. This correlation was significant only in participants who were insulin resistant at baseline.

**Conclusion:**

Within 24 hours of acute salt depletion, markers of insulin resistance exhibit a significant increase, which strongly correlates with change in IsoLG formation in APCs. This finding implies that oxidative stress in APCs may be implicated in the salt-sensitive modulation of glucose metabolism.

Insulin resistance and hypertension are closely linked as 2 hallmark components of metabolic syndrome. Salt sensitivity, defined by blood pressure changes in response to dietary sodium intake, is prevalent in approximately half of hypertensive people. Salt sensitivity is also strongly associated with insulin resistance (1), independent of confounding factors, including age and obesity (2). Furthermore, greater salt sensitivity correlates with more severe insulin resistance, suggesting a causal link. However, the pathogenetic link between the two remains elusive.

Insulin resistance and salt-sensitive hypertension are characterized by systemic inflammatory activation (3-5). Adipose tissue macrophages play a key role in obesity-induced insulin resistance as a major source of local and systemic inflammatory cytokines including tumor necrosis factor α (TNF-α), interleukin (IL)-1β, and IL-6 (6). These cytokines, in turn, impair insulin signaling at the insulin receptor level in target tissues including adipose tissue, skeletal muscle, and pancreas (7). Circulating concentrations of inflammatory markers negatively associate with insulin resistance and skeletal muscle oxidative capacity (6, 8), further highlighting the potential key role of inflammation in impaired insulin response.

We previously found that sodium enters antigen-presenting cells (APCs) and leads to the production of isolevuglandins (IsoLGs), highly reactive products of lipid peroxidation that rapidly adduct to proteins. Accumulation of IsoLG-adducts activates APCs and causes immune cell activation, resulting in systemic inflammation in salt-sensitive hypertension (3-5, 9). In previous clinical studies, IsoLG + APCs acutely decreased from salt loading to depletion, a phenomenon observed differentially in salt-sensitive people (10). It remains unclear whether salt-induced, IsoLG-mediated immune activation may play a role in the interplay between salt-sensitive hypertension and insulin resistance.

In this study, we investigated whether acute changes in salt induce changes in homeostatic model assessment for insulin resistance (HOMA-IR), a widely used biomarker of insulin resistance, and whether such change associates with salt-induced changes in IsoLG + APCs via a rigorous inpatient protocol of salt loading and depletion. We further assessed the differences in acute salt-induced changes between insulin-sensitive and insulin-resistant people.

## Methods

### Study Population

This study was designed as a prospective, interventional clinical trial with a single-group assignment. Participants were not randomized, and the study did not include a control group or placebo. Participants were recruited at the Vanderbilt University Medical Center (VUMC) between 2020 and 2024 through Researchmatch.org, VUMC email distribution lists, and flyers posted in outpatient clinics. Participants aged 18 to 65, either taking antihypertensive therapy or with systolic blood pressure (SBP) above 140 mmHg or diastolic blood pressure (DBP) above 90 mmHg, were included. Exclusion criteria included diabetes mellitus, confirmed or suspected renal, renovascular, or endocrine causes of secondary hypertension based on chart review of laboratory and imaging data and clinical documentation, and current treatment with agents known to increase blood pressure (eg, adrenergic agonists for attention deficit hyperactivity disorder, selective serotonin reuptake inhibitors, and serotonin and norepinephrine reuptake inhibitors; chronic use of decongestants or nonsteroidal anti-inflammatory drugs). In addition, individuals treated with agents known to modulate immune response (eg, glucocorticoids, immunosuppressants, direct immunomodulators), those with current excessive alcohol or illicit drug use, those with active or ongoing infectious or inflammatory disease (ie, active infection or connective tissue disorder), those with active or ongoing cancer, or a history of an acute cardiovascular event within 6 months of the study, as well as pregnant women were excluded. Demographic and clinical data were gathered from the participants and through chart review. The study was approved by the Institutional Review Board, and all subjects provided written informed consent prior to participation. The study was registered at Clinical Trials (NCT03753204).

### Inpatient Study Protocol

An inpatient protocol was used to assess the effects of salt loading and depletion, as described in previous publications (10, 11). During the screening visit, participants underwent a physical examination and had their blood pressure measured. All antihypertensive medications were discontinued at least 2 weeks prior to the study visit, and participants were instructed to maintain their regular diet during this period. For safety, blood pressure (BP) was measured twice daily in subjects who had stopped taking antihypertensive medications.

**Figure 1.**
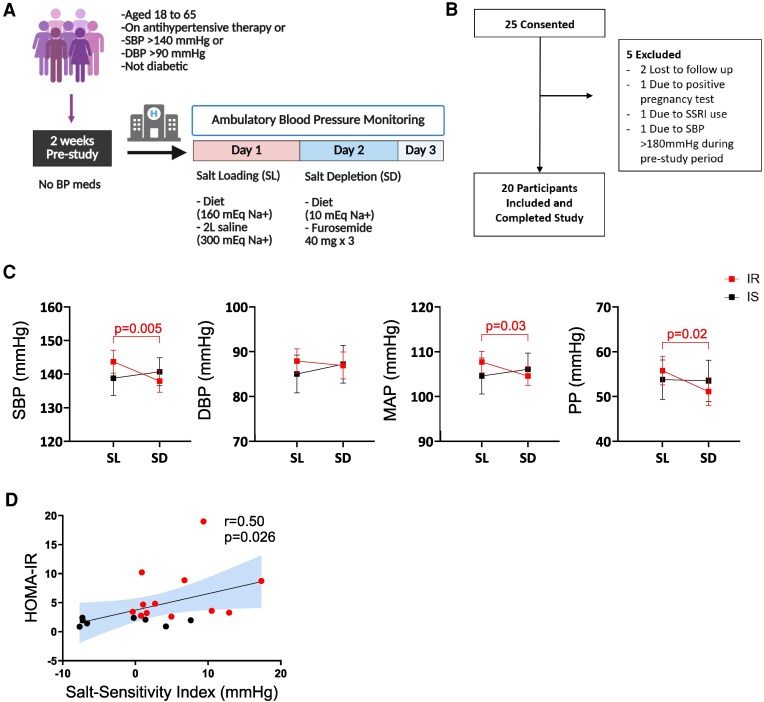
(A) The study protocol. (B) Participant recruitment flow chart. (C) Changes in systolic blood pressure (SBP), diastolic blood pressure (DBP), mean arterial pressure (MAP), and pulse pressure (PP) at salt loading and salt depletion. (D) The correlation of baseline HOMA-IR with salt sensitivity index. Data presented as mean and standard error of means (SEM). Figure 1A was created in BioRender. Ertuglu, L. (2025) https://BioRender.com/rnxg4rn.

Subsequently, participants were admitted to the VUMC Clinical Research Center for a 3-night stay to assess salt sensitivity using an inpatient protocol of salt loading and depletion, as shown in Fig. 1A. On the evening of admission, participants were given a regular dinner and instructed to rest. The next morning, BP recording began using ambulatory BP monitors (Spacelabs 90207). Baseline blood samples were collected at 8 Am before any interventions. On day 1, salt loading was conducted with a diet containing 160 mEq NaCl and 2L intravenous infusion of normal saline administered from 8 Am to 12 Pm.

The effects of salt depletion were examined on the following day (day 2). Blood samples were taken again at 8 Am to capture the salt-loaded state. Salt depletion was induced using 3 doses of oral furosemide 40 mg (administered at 8 Am, 12 Pm, and 4 Pm) and a diet containing 10 mEq NaCl. The third set of blood samples was collected at 8 Am on day 3 to reflect the salt-depleted state.

Throughout the study, participants had unrestricted access to water, but their food intake was limited to the diet specified by the protocol. Body weights were recorded at baseline and daily at 7 Am before any interventions. Body mass index (BMI) was calculated by dividing weight in kilograms by height in meters squared.

BP and pulse rate were recorded every 15 minutes during the day from 6 Am to 10 Pm and every 30 minutes at night throughout the 3-day study. The average BP recordings from 6 Am to 8 Am on day 1 were used as baseline BPs. The BPs recorded between 12 Pm to 10 Pm on days 1 and 2 were used to calculate the average BPs during salt loading and depletion, respectively.

### Flow Cytometry and Laboratory Analysis

Cell viability was assessed using the Zombie NIR™ Fixable Viability Kit (BioLegend, Cat# 423106). Fluorophore-conjugated antibodies were utilized at a concentration of 1 to 2 μg/100 μL, including Pacific Orange anti-CD45 (ThermoFisher, Cat# MHCD4530), PE-Cy7 anti-MerTK (BioLegend, Cat# 367610), Brilliant Violet 610 anti-HLA-DR (BD Biosciences, Cat# 562845), Brilliant Violet 711 anti-CD1c (BioLegend, Cat# 331536), Alexa Fluor 700 anti-CD14 (BioLegend, Cat# 367114), and Pacific Blue anti-CD16 (BioLegend, Cat# 980106). IsoLG protein adducts with any peptide backbone were identified using intracellular staining with the D11 ScFv antibody (12) (RRID: AB_ 3716551, https://www.antibodyregistry.org/update/3716551). After surface staining and cell fixation, a cell permeabilization kit (Invitrogen, Cat# GAS004) was employed to permeabilize the cells for intracellular detection of IsoLGs. For each experiment, we gated on live, single cells and then based on flow minus one control for each fluorophore to establish gates. Monocytes were categorized as classical (CD14^+^CD16^−^), intermediate (CD14^+^CD16^+^), or nonclassical (CD14^−^CD16^+^), while dendritic cells were identified as CD1c^+^. Data analysis was conducted using SpectroFlo^®^ Software (Cytek^®^ Biosciences).

Laboratory data, including blood counts, chemistries with electrolytes and creatinine, plasma glucose, and insulin were analyzed at the VUMC Pathology Laboratory.

### Statistical Analysis

Baseline data are expressed as median and interquartile range for continuous variables, and as frequencies and percentages for categorical variables. Unless specified otherwise, the analysis utilized the baseline percentages of immune cells containing IsoLGs and the changes in these percentages in response to salt loading or salt depletion. There was 1 participant who was missing IsoLG data, and 1 participant missing insulin data from day 3 (SD). Missing data was assumed to be missing at random and given the small proportion of missing data, complete case analysis approach was utilized.

The Wilcoxon rank-sum test was employed to compare measurements between salt loading and salt depletion. Since the baseline sodium intake of the participants were unknown, a comparison between baseline and salt loading or depletion was not carried out. Associations of interest involving continuous variables were assessed using Spearman's rank correlation. Trend lines and CIs were estimated using linear regression. A *P* value of .05 was set as the threshold for rejecting the null hypothesis. The analyses were conducted using Stata software (Stata/IC version 16).

## Results

### Baseline Characteristics

A total of 25 participants were consented and 20 completed the study (Fig. 1B). Table 1 shows the characteristics of the study population. Among the 20 participants, the mean (± standard error of means [±SEM]) age was 54 ± 1.8, with 12 female and 10 Black participants. Mean BMI was 31.2 ± 1.4 kg/m^2^. HOMA-IR was 4.5 ± 1.0 at baseline. All participants had normal serum creatinine levels at baseline. The salt sensitivity was quantified with salt sensitivity index (SSI), calculated as the change in SBP from salt loading to salt depletion. A positive SSI indicates a reduction in BP from salt loading to salt depletion. The mean SSI was 2.6 ± 1.5 mmHg.

**Table 1.** Baseline characteristics of the study population

Characteristic	All (n = 20)	Insulin sensitive (n = 8)	Insulin resistant (n = 12)	*P* value
Age, years	56.00 (49.50-60.00)	59.00 (51.50-60.00)	53.00 (49.50-59.00)	.37
Female sex, n (%)	12 (60)	6 (75)	6 (50)	.26
Black race, n (%)	10 (50)	7 (87.5)	3 (25)	.006
Weight, kg	91.65 (79.60-98.15)	84.65 (73.40-100.15)	93.40 (83.35-98.15)	.4
BMI, kg/m^2^	31.12 (29.13-33.80)	29.13 (23.51-37.50)	31.39 (30.35-33.80)	.31
SBP, mmHg	139.00 (127.45-146.50)	141.66 (128.01-154.44)	136.24 (127.45-145.95)	.62
DBP, mmHg	87.08 (80.98-96.06)	87.08 (83.98-93.15)	89.69 (76.60-96.56)	.7
MAP, mmHg	105.23 (98.82-114.11)	107.65 (98.75-115.25)	103.60 (98.90-110.55)	.85
PP, mmHg	46.06 (40.38-65.27)	49.26 (43.52-61.50)	44.26 (38.80-65.27)	.35
Fasting glucose, mg/dL	88.00 (84.00-94.00)	83.00 (80.50-87.50)	90.50 (86.00-105.00)	.01
Fasting insulin, uIU/mL	13.47 (9.68-18.75)	8.96 (5.85-10.29)	16.50 (14.73-36.74)	<.0001
HOMA-IR	2.98 (2.02-4.75)	1.95 (1.16-2.23)	4.13 (3.24-8.80)	<.0001
Serum Na, mmol/L	139.00 (138.50-140.50)	139.00 (137.50-139.50)	140.00 (139.00-141.00)	.25
Serum K, mmol/L	4.10 (3.80-4.40)	4.25 (3.85-4.55)	4.05 (3.70-4.40)	.46
Urinary Na+ excretion, mmol/day	115.70 (86.94-173.04)	101.44 (61.90-146.10)	117.32 (94.69-195.53)	.14

Data are presented as n (%) or median and interquartile range (IQR).

Abbreviations: BMI, body mass index; DBP, diastolic blood pressure; HOMA-IR, homeostasis model assessment index; MAP, mean arterial blood pressure, PP, pulse pressure; SBP, systolic blood pressure.

Among the participants, 12 had baseline HOMA-IR above 2.5 and therefore were classified as insulin resistant (13, 14). Among insulin-resistant participants, mean HOMA-IR was 6.3 ± 1.4 (Table 1). Baseline SBP and BMI were similar between insulin-sensitive and insulin-resistant groups.

### Relationship Between Baseline HOMA-IR and Salt Sensitivity

Figure 1C shows the mean blood pressures of insulin-sensitive and insulin-resistant participants at salt loading, and depletion. There was no significant difference between baseline SBP or DBP between insulin-sensitive and insulin-resistant participants (*P* = .6 and .7 for SBP and DBP, respectively). Among insulin-resistant participants, SBP significantly decreased from salt loading to depletion (*P* = .03). Similar changes were observed in pulse pressure (PP) among insulin-resistant participants. There was no significant change in SBP, DBP, mean arterial pressure (MAP), or PP among insulin-sensitive participants from salt loading to depletion (Fig. 1C).

Insulin-resistant participants were significantly more salt-sensitive than insulin-sensitive participants. The mean SSI (±SEM) was 5.7 ± 1.6 and −2.0 ± 2.1 mmHg for insulin-resistant and sensitive participants, respectively (*P* = .025). Among all participants, there was a significant positive correlation between baseline HOMA-IR and salt sensitivity (Fig. 1D).

### Effects of Acute Salt Loading and Depletion on HOMA-IR

**Figure 2.**
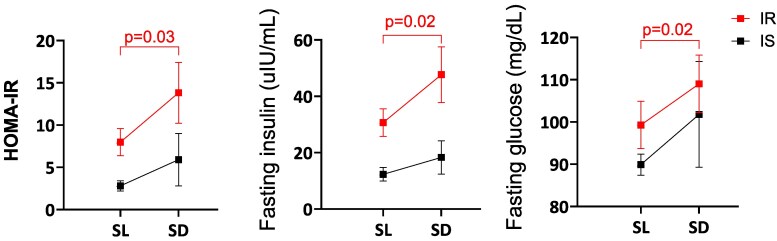
Changes in HOMA-IR, fasting insulin, and fasting glucose at salt loading, and salt depletion.

The mean (±SEM) HOMA-IR increased from 5.9 ± 1.13 to 10.91 ± 2.65 from salt loading to depletion among all participants (*P* = .011). Similarly, fasting insulin increased from 23.3 ± 3.7 to 36.9 ± 7.3 uIU/mL, and fasting glucose increased from 95.5 ± 3.6 to 106.1 ± 6.3 mg/dL (*P* = .009 and .037, respectively). In subgroup analysis, the change in HOMA-IR, fasting insulin, and fasting glucose from salt loading to depletion was significant only among insulin-resistant participants (*P* = .03, *P* = .02, and *P* = .02, respectively) (Fig. 2).

### Sodium-Induced Changes in Insulin Resistance Correlate With Changes in IsoLGs

**Figure 3.**
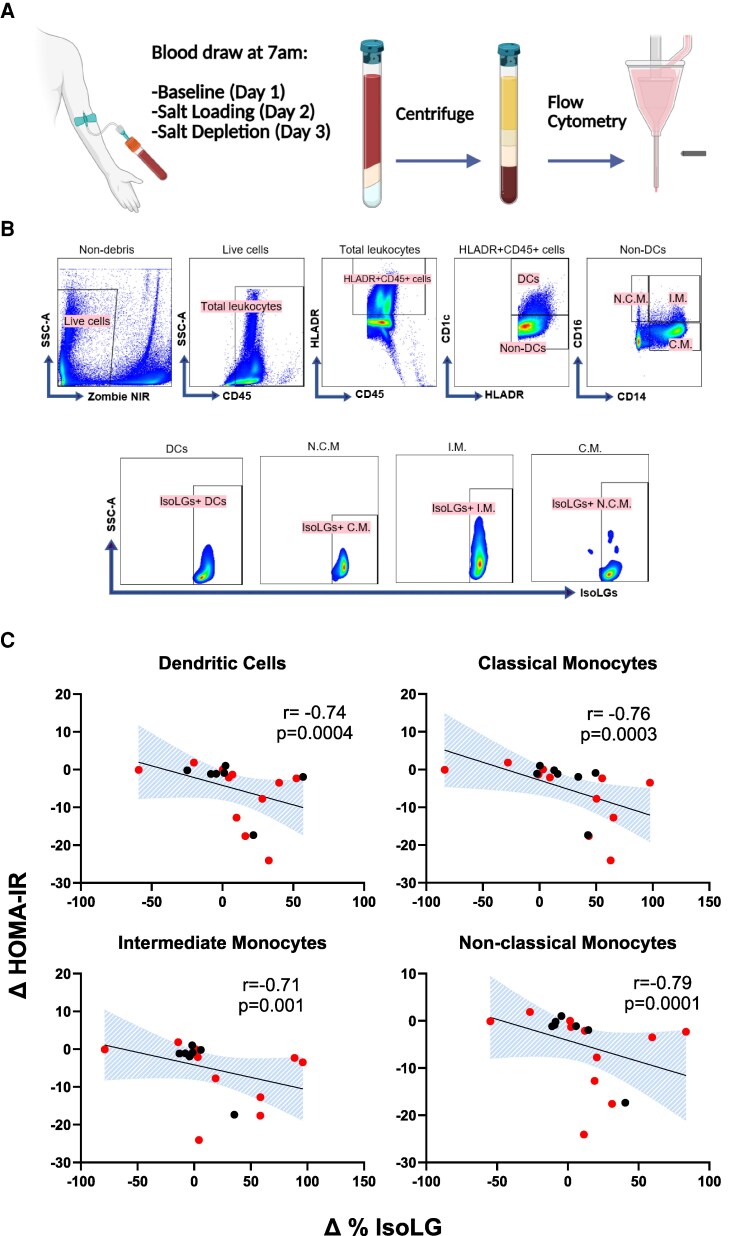
(A) The flow cytometry protocol. (B) Gating strategy for delineating IsoLG-adducts in dendritic cells (DC), nonclassical monocytes (N.C.M.), intermediate monocytes (I.M.) and classical monocytes (C.M.). (C) Correlations of change in HOMA-IR with changes in percent of IsoLGs in APCs from salt loading to salt depletion (SL-SD). Data presented as mean and standard error of means (SEM). Figure 3A was created in BioRender. Ertuglu, L. (2025) https://BioRender.com/zldu97z.

To determine whether there is an association between salt-induced changes in IsoLGs and salt-induced changes in HOMA-IR, the percentage of IsoLG + APCs was measured at baseline, after salt loading, and salt depletion with flow cytometry. Figure 3 depicts the relationship between changes in the percent of IsoLG + APCs and HOMA-IR. Salt-induced changes in HOMA-IR correlated positively with changes in IsoLGs in all APCs, such that participants with a greater increase in insulin resistance from salt loading to depletion had a greater decrease in IsoLGs. In subgroup analysis, this correlation remained significant only among insulin-resistant participants (Table 2).

**Table 2.** Correlations of change in HOMA-IR with changes in percent of IsoLG + APCs from salt loading to salt depletion (SL − SD) in insulin-resistant vs insulin-sensitive group

	Insulin resistant		Insulin sensitive	
Correlation with ΔHOMA-IR	R	*P* value	R	*P* value
DC	−0.71	.018	−0.46	.30
CD14+	−0.76	.009	−0.46	.30
CD14+16+	−0.67	.028	−0.40	.96
CD16+	−0.63	.039	−0.50	.25

## Discussion

In this study, we found that acute salt depletion leads to significant increases in fasting glucose, insulin, and HOMA-IR in insulin-resistant but not insulin-sensitive people with hypertension. The change in HOMA-IR from salt loading to depletion significantly correlated with changes in IsoLG-adduct accumulation in APCs. In subgroup analysis, this correlation was significant only in the insulin-resistant group, suggesting a role of salt-induced, IsoLG-mediated inflammation in insulin resistance.

We observed a strong correlation between SSI and HOMA-IR, which corroborate with previous findings of several studies (15-18). Growing evidence from clinical studies suggests that a low salt diet leads to an increase in insulin resistance, assessed by HOMA-IR, euglycemic hyperinsulinaemic clamp study (19), and an increase in glycemic response in the oral glucose tolerance test (20). While salt restriction typically triggers a physiological increase in the renin-angiotensin system, the impact of salt restriction on insulin sensitivity was found to be independent of activation of this system (21-23), implying a direct effect of salt restriction on insulin signaling mechanisms (23). The current study is the first to explore the relationship between inflammatory activation and insulin sensitivity in response to acute changes in salt intake.

Inflammation can influence insulin resistance through several mechanisms. Obesity-related insulin resistance is marked by increased macrophages in the adipose tissue, which is the key source of local and systemic secretion of pro-inflammatory cytokines TNF-α, IL-1β, and IL-6 (6). By activating various kinases, such as c-Jun N-terminal kinase, protein kinase C, inducible nitric oxide synthase, which in turn phosphorylate insulin receptors and insulin receptor substrates, these cytokines inhibit insulin signaling in target tissues, including liver and skeletal muscle (7, 24-26). Salt-sensitive hypertension is also strongly linked with inflammation (27). We previously demonstrated that sodium enters dendritic cells via amiloride-sensitive epithelial sodium channels (ENaC) and leads to intracellular calcium influx which in turn activates protein kinase C and NADPH oxidase, resulting in IsoLG formation (9, 28). IsoLGs activate NLRP3 inflammasome, resulting in IL-1β production, and stimulate T cells to generate pro-hypertensive cytokines IL-6, IL-17A, and TNF-α (11). In clinical studies, higher IsoLG-adduct accumulation in APCs correlates with higher salt sensitivity, supporting a key role of salt-induced IsoLG-dependent APC activation in the pathogenesis of salt sensitivity. In this study, we found initial evidence that salt-induced changes in insulin sensitivity are linked with acute changes in IsoLG-adducts in APCs. It should be noted that in the current study, changes in IsoLG-adducts in circulating APCs were measured, which may be the result of ENaC-dependent sodium entry to APCs but also of general manifestation of increased oxidative stress. Nevertheless, these results suggest that salt-induced changes in IsoLG formation in APCs may be implicated in the interplay between salt-sensitive hypertension, insulin resistance, and systemic inflammation. Importantly, this relationship was evident only among people with baseline insulin resistance. An impairment in insulin sensitivity may be required to observe an additional effect of salt-induced IsoLG and downstream immune activation on insulin sensitivity.

Previous research investigating the impact of salt on insulin sensitivity has typically noted changes over a period of 5 to 7 days (21, 23, 29, 30). In this study, we observed significant changes in fasting insulin, glucose, and HOMA-IR occur within just 24 hours of salt depletion. This rapid response underscores the dynamic nature of salt-induced changes in insulin metabolism, particularly among insulin-resistant individuals.

This study has several limitations. Firstly, the sample size was relatively small, and the study was exploratory in nature. It is noteworthy that insulin-sensitive group had significantly more Black participants than the insulin-resistant group; therefore, a possible effect of racial differences and whether the current findings are generalizable needs to be investigated in future studies. Furthermore, the study population consisted only of hypertensive individuals; thus, it remains unclear whether such associations apply to normotensive individuals. Additionally, both salt sensitivity and insulin resistance have strong associations with aging. The current study included mostly middle-aged adults with limited variation in age, therefore it was not possible to assess a potential effect of age. The study design was cross-sectional, and the findings are not adequate to infer direct causality. Further research with larger sample sizes is necessary to expand upon these findings.

The major strength of the study is the use of a rigorous inpatient salt loading and depletion protocol, which enables close control of dietary intake and limits variability in confounding factors.

In conclusion, we have identified that HOMA-IR, a surrogate of insulin resistance, acutely increases from salt loading to salt depletion, which correlates with changes in IsoLG formation in circulating APCs. The findings suggest that increased IsoLG formation may play a role in the inflammatory processes interlinking insulin resistance and salt-sensitive hypertension.

## Summary Table

What is known about the topic:

- Insulin resistance is linked to salt sensitivity and low sodium intake.
- Sodium-induced IsoLG formation in antigen-presenting cells leads to systemic inflammation and salt-sensitive hypertension.
- Acute changes in salt intake result in changes in IsoLG formation in APCs in salt-sensitive people.

What this study adds:

- Acute salt depletion within 24 hours significantly increases markers of insulin resistance.
- Changes in insulin resistance with acute salt depletion are strongly correlated with changes in IsoLG formation in APCs.
- This correlation is significant only in participants who are insulin resistant at baseline.

## Data Availability

The data that support the findings of this study are available from the corresponding author, AK, upon reasonable request.

## Clinical Trial Information

Clinicaltrials.gov registration: NCT03753204.

## Abbreviations

APC: antigen-presenting cell
BMI: body mass index
BP: blood pressure
DBP: diastolic blood pressure
ENaC: epithelial sodium channel
HOMA-IR: homeostatic model assessment for insulin resistance
IL-: interleukin
IsoLG: isolevuglandin
MAP: mean arterial pressure
PP: pulse pressure
SBP: systolic blood pressure
SEM: standard error of means
SSI: salt sensitivity index
TNF-α: tumor necrosis factor alpha
VUMC: Vanderbilt University Medical Center
